# Living well with dementia: An exploratory matched analysis of minority ethnic and white people with dementia and carers participating in the IDEAL programme

**DOI:** 10.1002/gps.6048

**Published:** 2024-01-05

**Authors:** Christina R. Victor, Laura D. Gamble, Claire Pentecost, Catherine Quinn, Catherine Charlwood, Fiona E. Matthews, Linda Clare

**Affiliations:** ^1^ Department of Health Sciences College of Health, Medicine and Life Sciences Brunel University London London UK; ^2^ Population Health Sciences Institute Newcastle University Newcastle upon Tyne UK; ^3^ University of Exeter Medical School University of Exeter Exeter UK; ^4^ Centre for Applied Dementia Studies Faculty of Health Studies University of Bradford Bradford UK; ^5^ University of Exeter Medical School University of Exeter, and NIHR Applied Research Collaboration South‐West Peninsula Exeter UK

**Keywords:** carers, dementia, minority ethnic population, quality of life

## Abstract

**Objectives:**

The increasing heterogeneity of the population of older people is reflected in an increasing number of people with dementia and carers drawn from minority ethnic groups. Data from the IDEAL study are used to compare indices of ‘living well’ among people with dementia and carers from ethnic minority groups with matched white peers.

**Methods:**

We used an exploratory cross‐sectional case‐control design to compare ‘living well’ for people with dementia and carers from minority ethnic and white groups. Measures for both groups were quality of life, life satisfaction, wellbeing, loneliness, and social isolation and, for carers, stress, relationship quality, role captivity and caring competence.

**Results:**

The sample of people with dementia consisted of 20 minority ethnic and 60 white participants and for carers 15 and 45 respectively. People with dementia from minority ethnic groups had poorer quality of life (−4.74, 95% CI: −7.98 to −1.50) and higher loneliness (1.72, 95% CI: 0.78–2.66) whilst minority ethnic carers had higher stress (8.17, 95% CI: 1.72–14.63) and role captivity (2.00, 95% CI: 0.43–3.57) and lower relationship quality (−9.86, 95% CI: −14.24 to −5.48) than their white peers.

**Conclusion:**

Our exploratory study suggests that people with dementia from minority ethnic groups experience lower quality of life and carers experience higher stress and role captivity and lower relationship quality than their white peers. Confirmatory research with larger samples is required to facilitate analysis of the experiences of specific minority ethnic groups and examine the factors contributing to these disadvantages.

## Key points

- We wanted to investigate if people with dementia and their carers from minority ethnic groups had worse living well outcome than their white British peers
- We used a novel method‐an exploratory cross sectional case control study‐ to investigate this question using data from the IDEAL cohort
- People with dementia from minority ethnic groups had poorer quality of life and higher levels of loneliness than their white British contemporaries
- Carers from minority ethnic groups demonstrated higher levels of stress and role captivity and lower relationship quality than their white counterparts
- Confirmatory research with larger samples is required verify the disadvantages experienced by minority ethnic people with dementia and their carers and examine the factors contributing to these

## 1 INTRODUCTION

Dementia is a significant public health challenge in terms of the population affected and disease burden. Current estimates suggest there are approximately 885,000 people living with dementia in the UK increasing to 1.6 million over the next 2 decades.^1^ It is a leading cause of mortality, accounting for 12.8% of deaths in England, 68,000 in 2019.^2^

Future populations of people aged 60 and over in Britain will be characterised by increasing diversity resultant from the ageing of the post war migrants from the Indian sub‐continent and Caribbean. Population projections for 2050 estimate that 27% of those aged 60 and older will be from minority ethnic groups compared with 5% in 2021.^3^ This suggests a fifteen‐fold increase in the number of minority ethnic elders with dementia from 26,000 to 400,000 by 2050. Research evidence about experiences of dementia among minority ethnic groups is sparse^4^ and focuses on three key topics: establishing the disease burden; investigating access to and quality of services including diagnosis; and understanding experiences of living with dementia for individuals and their care partners.

Within the UK, a limited number of studies have reported dementia prevalence/incidence in minority ethnic groups and compared this with white peers. A review of three cross‐sectional community‐based surveys of African‐Caribbean (AC) elders reported higher dementia prevalence in this population independent of dementia assessment measures used^5^ (Comprehensive Assessment and Referral Evaluation; Short‐CARE^6^ (AC v reference population: 17% v 10%); MMSE Mini Mental State Examination; MMSE^7^ ((34% v 4%) and GMS‐AGECAT (Geriatric Mental State Examination algorithm—GMS‐AGECAT^8^ (8% v 3%). A matched general practice study in London reported a significantly higher prevalence of dementia, as defined by a diagnostic interview, for AC elders of 9.6% compared with 6.9% for their white counterparts (odds ratio of 3.1, 95% CI 1.3–7.3, *p* = 0.012 after adjustment for age and socioeconomic status).^9^ A study using the UK Biobank reported a higher risk of dementia for Black participants (hazard ratio of 1.63 95% CI 1.22–2.19 *p* = 0.002), after adjustment, compared with white participants but no difference for Asian participants.^10^

The UK National Dementia Strategy^11^ emphasised the importance of getting a diagnosis of dementia, as this is the start of the pathway to treatment and services and can provide details about dementia incidence/prevalence and service access across different populations.^12^ UK prevalence estimates based upon a recorded diagnosis suggest that Black populations have higher risk of dementia than their white or South Asian peers. A matched study in east London reported that a recorded diagnosis of dementia was more likely for older people of Black (OR 1·43, 95% CI 1·31–1·56) and South Asian (OR 1·17, 95% CI 1·06–1·29) ethnicities relative to their white peers.^13^ Similar findings are reported using electronic primary care records for England. Compared with the white British population, recorded dementia diagnosis was higher for the Black population (25% for women and 28% for men) and lower for Asian ethnic groups (18% for women and 12% for men).^14^ UK Biobank data demonstrate that dementia risk over a 14 year period defined as either self‐reported or clinical records, was highest for Black participants (hazard ratio 1.43, 95% CI 1.16–1.77, *p* = 0.001)^15^ and remained so after adjustment for 12 modifiable risk factors (HR 1.34, 95% CI 1.06–1.69, *p* = 0.014). Measuring disease burden by diagnostic and referral records excludes those not accessing primary care, underrepresents people living in deprived areas and relies upon the recording of ethnicity in medical records, which may be incomplete. For example, in an electronic records study ethnicity was not recorded in 55% of records for those with a dementia diagnosis.^14^ In the Biobank study there were 294,162 participants of whom 1.2% (3590) were South Asian and 0.9% (2766) Black. Of the 5972 incident dementia cases 96% (5789) were white, 1.3% (79] South Asian and 1.5% (91) Black.^15^ Research participation from minority ethnic groups remains challenging.^16^, ^17^

A Nottingham based study reported no difference in timely access to dementia assessments, defined as within 90 days of referral, for white and South Asian patients.^18^ A survey of referrals to memory assessment clinics in England concluded that non‐white participants presented with lower cognitive function scores than white participants.^19^ Primary care records from Bristol concluded that people from minority ethnic groups were less likely to receive a cognitive assessment, and scored less well when assessed, than their white peers.^20^

These inequalities in the dementia disease burden borne by minority ethic elders of later diagnosis, less access to care and/or culturally appropriate care are replicated internationally.^21^ Observational studies from the United States for those aged 65 and over reported that African Americans had the highest prevalence of dementia followed by Hispanic elders and the white non‐Hispanic population had the lowest prevalence.^22^, ^23^ Within Europe a review of 7 studies, 5 from the UK and 1 each from Norway and the Netherlands, concluded that there was an excess risk of dementia for African Europeans (OR 1.82; CI 1.31–2.53) and Asian Europeans (OR 2.10 95% CI 1.21–3.67).^24^ Underdiagnosis of dementia is evident in a range of European countries. A study from Demark suggested that only 11% of the expected number of older people from minority ethnic groups with dementia received a diagnosis.^25^ Alzheimer's USA reports the discrimination experienced by minority elders seeking dementia care.^26^

For those living with dementia, the support of family and friends is key to remaining in the community and promoting wellbeing. It is estimated that there are approximately 580,000 carers supporting people living with dementia in the UK.^27^ Extrapolating from this, approximately 17,500 are drawn from minority ethnic communities and this will increase in coming years. Qualitative studies have reported the experiences of caregiving for people with dementia from minority ethnic groups have reported the experiences of Black African and Caribbean people in the UK and USA, Asian groups in the UK and a diverse range of minority groups across Europe.^28^ Quantitative research comparing the experiences of carers from different minority ethnic groups or comparing outcomes such as quality of life or life satisfaction is limited. In the review of 38 studies on caregiving for people with dementia from minority groups in the USA, 2 reported life satisfaction, 1 general wellbeing and 1 spiritual wellbeing. Two studies reported higher wellbeing for African American caregivers compared with their white counterparts.^29^ A survey of carers in England did not find a consistent relationship between health‐related quality of life, as measured by EQ‐5D scores, and ethnicity.^30^ Carers of those referred to memory clinics in England did not show variations in quality of life by ethnicity using EG‐5D‐3L.^31^

There is a clear evidence gap enumerating and comparing outcomes such as quality of life or wellbeing for people with dementia and their carers from different ethnic groups with their white peers. We use data from the IDEAL programme to undertake an exploratory analysis compare ‘living well’ among people with dementia and carers from ethnic minority groups with that of their matched white peers.^32^ Our rationale for using the term living derives from the voices of people with dementia themselves. It reflects their arguments that we reframe our conceptualisation of dementia from ‘a living death’ to one that focuses upon promoting and supporting opportunities to live fulfiling lives.^33^ Indeed this ambition is reflected in the title of the national dementia strategy, ‘Living Well with Dementia’.^11^

## 2 MATERIALS AND METHODS

### 2.1 Study population

Our analysis uses data from the IDEAL programme: a longitudinal study comprising 1537 people at Time 1 (T1) recruited between 2014 and 2016 with subsequent follow up at 12 (T2) and 24 (T3) months.^32^ Participants were recruited through a network of 29 National Health Service sites in England, Scotland, and Wales. Inclusion criteria were: living in the community; a diagnosis of any type of dementia; and a Mini‐Mental State Examination^7^ score ≥15, indicative of mild‐to‐moderate dementia.^32^ Where the person with dementia was willing, their caregiver was also approached to participate in the study. At T1 1277 carers took part. In 2018, 2 years after T3, the cohort was followed up annually for a further 3 years (T4–T6) and an enrichment sample recruited to enhance the initial cohort by inclusion of specific groups: those with rare dementias, undiagnosed dementia or living alone.^34^ We include all minority ethnic participants from both the baseline and enrichment cohorts based upon participants' self‐identification as a member of one of 18 different groups using a standard question routinely used in the UK.

### 2.2 Analytical approach

We employed a cross‐sectional case‐control design to compare ‘living well’ for people with dementia and carers from minority ethnic and white groups using two matched samples to explore differences in outcomes based on self‐reported ethnicity identity. There were 20 people with dementia from minority ethnic groups, and 15 carers. Given the small number of participants we were unable to undertake the analysis by specific ethnic groups but created a single grouping using the approach of Dodd and colleagues.^20^ People of white ethnicity were matched 3:1 to people from minority ethnic groups. For people with dementia this was based on age group, sex, dementia subtype, time since diagnosis, area‐level deprivation, and residence in an urban or rural location (see supplementary figure S1 for an overview of the matching procedure). For carers, this was based on age group, sex, dementia subtype of the person with dementia, kin relationship to the person with dementia, living situation (living alone or with others), hours spent providing care, area‐level deprivation, and residence in an urban or rural location (see supplementary Figure S1). This generated a sample of 20 people with dementia from minority ethnic groups matched with 60 of white ethnicity, and 15 carers from minority ethnic groups matched with 45 of white ethnicity. The analysis was conducted using version 7 of the dataset.

### 2.3 Key measures

Key measures used to profile our sample of people with dementia are sex and age of the person with dementia (<65, 65–69, 70–74, 75–79, 80+), dementia subtype dementia subtype ‐ Alzheimer's disease (AD), vascular dementia (VaD), mixed Alzheimer's and vascular dementias (mixed AD/VaD), frontotemporal dementia (FTD), dementia with Lewy bodies (DLB), Parkinson's disease dementia and other/unspecified, time since diagnosis (<1 year, 1–2 years, 3–5 years, 6+ years), marital status (married vs. single/divorced/widowed) and living situation (living alone vs. living with others). Diagnosis and time since diagnosis were obtained from clinical records. Two area‐level measures of deprivation were used in the matched sample analysis: the Index of Multiple Deprivation and whether the person with dementia lived in an urban or rural location.^32^ Three deprivation quantiles were used for analysis (Q1—most deprived, Q3—least deprived).

For the carers key measures were sex and age (<65, 65–69, 70–74, 75–79, 80+), dementia subtype of the care recipient (AD, VaD, mixed AD/VAD or other) relationship to the person with dementia (spouse/partner or family/friend), living situation (lives with or does not live with the person with dementia), hours spent providing care per day (less than 1 h, 1–10 h, or more than 10 h), area‐level deprivation and whether they live in an urban or rural area.

We have argued that ‘living well’ is a multifaceted concept that is a continuum^33^ operationalised in our cohort by three indicators: quality of life, life satisfaction and wellbeing. For people with dementia, quality of life was measured with the QoL‐AD scale (score range 13–52)^35^; higher scores indicate better quality of life. For carers, quality of life was measured using the World Health Organization QoL‐BREF (WHOQOL‐BREF).^36^ As this measure does not yield a total score, a factor analysis was conducted to estimate factor scores for those with complete data. Further ‘living well’ measures and measures of social connection were completed by both the person with dementia and carers. Satisfaction with life was measured using the Satisfaction with Life Scale (SwLS; score range 5–35)^37^; higher scores indicate better satisfaction with life. Well‐being was measured with the World Health Organization‐Five Well‐being Index percentage score (WHO‐5; score range 0–100).^38^ There were two measures of social connection. Loneliness was measured using the six‐item De‐Jong Gierveld Loneliness Scale (range 0–6, high scores indicate greater loneliness).^39^ Social isolation was measured using the six‐item Lubben Social Network Scale (range 0–30)^40^; higher scores indicate less social isolation.

Carers completed several additional measures. Stress was assessed with the Relative Stress Scale (range 0–60)^41^; a higher score indicates greater stress. Short, standardized measures assessed caring role captivity^42^ and competence.^43^ Scores for both measures ranged from 3 to 12, with higher scores indicating greater caregiving role captivity or greater competence. Current relationship quality between carers and their care partner was assessed using the Positive Affect Index.^44^ Scores range from 5 to 30, with higher scores indicating better relationship quality.

### 2.4 Statistical analyses

For both people with dementia and carers, we compared scores for quality of life, satisfaction with life, wellbeing, loneliness, and social isolation for the minority and white ethnic groups. In addition, for carers, we compared relationship quality, caregiver stress, competence, and role captivity. Mean scores for each group were reported, and regression, adjusted for matching variables and cohort (original or enrichment), undertaken. Residuals were checked for normality and linear regression models conducted for wellbeing, satisfaction with life, quality of life, social network and caregiver stress. Quantile regression (median) was conducted for loneliness, relationship quality, caregiver competence and role captivity. Missing data on outcomes and covariates was imputed using multiple imputation with chained equations. Estimates were combined according to Rubin's rules.^45^ Analyses were conducted in Stata 16.

## 3 RESULTS

*Sample characteristics*: The matched sample of people with dementia included 20 participants who self‐identified as having Indian/Pakistani/Bangladeshi (*N* = 9), black African or Caribbean (*N* = 8) or mixed (*N* = 3) ethnic identity and 60 white participants (see Table 1). For the carers sample we had 15 participants self‐identify as Indian/Pakistani/Bangladeshi (*N* = 9); Black African/Caribbean (*N* = 2) or mixed ethnicity (n = 4) and 45 white (see supplementary Table S1 for full details of the ethnic minority sample). Our samples of ethnic minority group carers and people with dementia included 9 dyads where both caregiver and care recipient were in the sample. However, for 6 minority carers the care partner identified as white British.

**TABLE 1:** Characteristics of the participant samples.

		White (*n* = 60)		Ethnic minority (*n* = 20)		Total cohort (*n* = 1741)	
		*N*	%	*N*	%	*N*	%
People with dementia							
Age group^a^	<65	12	20.0	4	20.0	199	11.4
	65–69	3	5.0	1	5.0	201	11.5
	70–74	9	15.0	3	15.0	293	16.8
	75–79	21	35.0	7	35.0	381	21.9
	≥80	15	25.0	5	25.0	667	38.3
Sex^a^	Men	48	80.0	16	80.0	1002	57.6
	Women	12	20.0	4	20.0	739	42.4
Dementia subtype^a^	Alzheimer's disease	33	55.0	7	35.0	910	52.3
	Vascular dementia	3	5.0	4	20.0	179	10.3
	Mixed (Alzheimer's and vascular)	15	25.0	6	30.0	347	19.9
	Frontotemporal dementia	6	10.0	2	10.0	99	5.7
	Dementia with Lewy bodies	3	5.0	1	5.0	73	4.2
	Parkinson's disease dementia	0	0.0	0	0.0	92	5.3
	Other/unspecified	0	0.0	0	0.0	41	2.4
Length of time since diagnosis^a^	<1 year	41	68.3	11	68.8	883	55.6
	1–2 years	10	16.7	3	18.8	508	32.0
	3–5 years	9	15.0	2	12.5	168	10.6
	6+ years	0	0.0	0	0.0	29	1.8
	Missing^b^	‐	‐	4	‐	153	‐
Deprivation^a^	1–most deprived	28	46.7	12	60.0	316	18.2
	2	18	30.3	5	25.0	629	36.1
	3–least deprived	14	23.3	3	15.0	796	45.7
Location^a^	Urban	56	93.3	19	95.0	1172	67.3
	Rural	4	6.7	1	5.0	569	32.7
Marital status	Married	53	90.0	18	90.0	1309	75.2
	Widowed/divorced/single	7	10.0	2	10.0	432	24.8
Living situation	Lives alone	8	13.3	2	10.0	290	16.8
	Lives with others	52	86.7	18	90.0	1449	83.2
	Unclassifiable	‐	‐	‐	‐	2	‐
Ethnicity	White	60	100.0	0	0.0	1696	98.8
	Bangladeshi, Indian and Pakistani	0	0.0	9	45.0	9	0.5
	Black‐African or Caribbean	0	0.0	8	40.0	8	0.5
	Mixed	0	0.0	3	15.0	3	0.2
	Missing	‐	‐	‐	‐	25	‐
IDEAL cohort	Enrichment	9	15.0	3	15.0	204	11.7
	Original	51	85.0	17	85.0	1537	88.3

		White (*n* = 45)		Ethnic minority (*n* = 15)		Total cohort (*n* = 1452)	
		*N*	%	*N*	%	*N*	%
Carers							
Age group^a^	<65	30	66.7	10	66.7	449	31.0
	65–69	6	13.3	2	13.3	238	16.4
	70–74	9	20.0	3	20.0	292	20.2
	75–79	0	0.0	0	0.0	236	16.3
	80+	0	0.0	0	0.0	232	16.0
	Missing	‐	‐	‐	‐	5	‐
Sex^a^	Men	15	33.3	5	33.3	429	29.5
	Women	30	66.7	10	66.7	1023	70.5
Dementia subtype of care recipient^a^	AD/VaD/Mixed	37	82.2	10	71.4	1193	82.2
	Other	8	17.8	4	28.6	259	17.8
	Missing	‐	‐	1	‐	‐	‐
Deprivation^a^	1–most deprived	24	53.3	8	53.3	234	16.2
	2	14	31.1	5	33.3	512	35.5
	3–least deprived	7	15.6	2	13.3	696	48.3
	Missing	‐	‐	‐	‐	10	‐
Hours of care^a^	Less than 1 h	9	20.0	9	20.0	311	21.8
	1–10 h	15	33.3	5	33.3	581	40.7
	More than 10 h	21	46.7	7	46.7	537	37.6
	Missing	‐	‐	‐	‐	23	‐
Living situation^a^	Lives with care recipient	33	73.3	11	73.3	1243	85.7
	Does not live with care recipient	12	26.7	4	26.7	207	14.3
	Unclassifiable	‐	‐	‐	‐	2	‐
Carer type^a^	Spouse/partner	33	73.3	11	73.3	1180	81.3
	Family or friend	12	26.7	4	26.7	272	18.7
Location^a^	Urban	39	86.7	13	86.7	965	66.9
	Rural	6	13.3	2	13.3	477	33.1
	Missing	‐	‐	‐	‐	10	‐
Ethnicity	White	45	100.0	0	1435	1435	98.9
	Bangladeshi, Indian and Pakistani	0	0.0	9	60.0	9	0.6
	Black‐African or Caribbean	0	0.0	2	13.3	3^c^	0.2
	Mixed	0	0.0	4	26.7	4	0.3
	Missing	‐	‐	‐	‐	1	‐
IDEAL cohort	Enrichment	10	22.2	4	26.7	175	12.1
	Original	35	77.8	11	73.3	1277	87.9

^a^ These variables were used in matching procedure for the white and ethnic minority groups.

^b^ The four people of ethnic minority that are missing length of time since diagnosis were not matched on this variable.

^c^ 1 person of ethnic minority in the overall sample was excluded from our analysis because of missing data on all outcome measures.

As intended our minority ethnic and white populations were broadly comparable (Table 1). For people with dementia, both groups demonstrated a predominance of male participants (80% across both matched groups), those aged 75 and over (60% across both matched groups), those married (90% ethnic minority matched group and 83% white matched group) and those diagnosed for less than a year approximately 68% across both groups). Approximately half of the white matched sample, 55%, had a diagnosis of AD, compared with 35% for the ethnic minority group. Similar comparability is characteristic of the two groups of carers.

*Living well measures*: Our descriptive analysis shows that for people with dementia from minority groups, scores on all three measures of ‘living well’, well‐being (WHO‐5), satisfaction with life (SwLS) and quality of life (QoL‐AD), were approximately 10% lower than their white peers (Table 2). In addition, both loneliness and isolation were higher among minority ethnic group participants compared with their white peers. For carers there were no differences between our two groups in ‘living well’ measures but loneliness, and caregiver strain, stress and competence were worse for minority group participants (Table 3).

**TABLE 2:** Outcomes for matched sample of people with dementia and comparison with total cohort.

Measure	White (*n* = 60)		Ethnic minority (*n* = 20)		Estimate (95% CI)^a^	Total cohort (*n* = 1741)	
	Mean (sd)	N	Mean (sd)	*N*		Mean (sd)	*N*
Wellbeing	61.1 (20.3)	60	52.2 (25.7)	19	−8.38 (−20.37–3.61)	60.3 (20.9)	1713
Satisfaction with life	26.0 (6.9)	60	24.6 (7.0)	19	−1.61 (−5.21–1.99)	25.8 (6.3)	1689
Quality of life	37.7 (6.0)	60	32.8 (8.1)	19	**−4.74 (−7.98–‐1.50)**	36.6 (6.1)	1567
Social network	16.3 (6.4)	60	13.3 (5.5)	18	−2.99 (−6.64–0.65)	15.2 (6.2)	1649
Loneliness	1.0 (1.2)	60	2.0 (1.9)	18	**1.72 (0.78–2.66)**	1.4 (1.5)	1629

*Note*: People with dementia.

^a^ Adjusted for matching variables (age, sex, diagnosis type, length of time since diagnosis, deprivation and rural or urban location) and cohort (original or enrichment).

Bold indicates that the 95% confidence intervals do not cross 0 (for estimates).

**TABLE 3:** Outcomes for matched sample of caregivers and comparison with total cohort.

Measure	White (*n* = 45)		Ethnic minority (*n* = 15)		Estimate (95% CI)^a^	Total cohort (*n* = 1452)	
	Mean (sd)	*N*	Mean (sd)	*N*		Mean (sd)	*N*
Wellbeing	52.6 (21.0)	45	51.5 (28.4)	15	−0.55 (−14.32–13.22)	55.2 (20.3)	1407
Satisfaction with life	21.5 (7.6)	45	19.9 (9.2)	15	−1.91 (−6.65–2.83)	23.6 (6.6)	1401
Quality of life	−0.24 (2.01)	45	−1.32 (3.26)	14	−1.25 (−2.67–0.16)	0.18 (2.10)	1380
Social network	16.8 (5.5)	45	13.9 (7.1)	15	−2.62 (−6.39–1.15)	17.5 (5.6)	1392
Loneliness	2.7 (1.9)	45	2.8 (2.5)	15	0.00 (−1.52–1.52)	2.5 (1.9)	1356
Current relationship quality	24.2 (3.6)	45	17.2 (7.0)	15	**−9.86 (−14.24–‐5.48)**	23.1 (4.8)	1410
Stress	21.1 (10.3)	45	29.5 (13.9)	13	**8.17 (1.72–14.63)**	19.6 (9.9)	1359
Caregiver competence	9.0 (1.6)	45	8.8 (2.9)	14	0.00 (−1.61–1.61)	9.1 (1.7)	1397
Role captivity	5.7 (2.4)	45	8.1 (2.8)	14	**2.00 (0.43–3.57)**	5.6 (2.3)	1394

*Note*: Study name for overall cohort removed for review.

^a^ Adjusted for matching variables (caregiver age, caregiver sex, diagnosis type of person with dementia, caregiver type, hours of care, living situation, deprivation and rural or urban location) and cohort (original or enrichment).

Bold indicates that the 95% confidence intervals do not cross 0.

Our regression analysis demonstrated that people with dementia from minority ethnic groups have a poorer quality of life (−4.74, 95% CI: −7.98 to −1.50) but not poorer wellbeing or satisfaction with life. In addition, people with dementia had significantly higher scores for loneliness (1.72, 95% CI: 0.78–2.66). The relationship with social isolation is unclear. Minority ethnic carers demonstrated significantly higher levels of stress (8.17, 95% CI: 1.72–14.63) and role captivity (2.00, 95% CI: 0.43–3.57) and lower relationship quality (−9.86, 95% CI: −14.24 to −5.48) than their matched white peers. There was no difference between the two groups in caregiver competence.

## 4 DISCUSSION

Using data collected as part of the IDEAL programme, we investigated ‘living well’ for people with dementia and carers from ethnic minority groups compared to matched white peers. Our key findings highlighted the significantly poorer quality of life and higher loneliness of minority ethnic people with dementia and significantly higher stress and role captivity and lower relationship quality for minority ethnic carers compared with their respective white peers.

It is important to acknowledge the limitations of our exploratory study. The IDEAL cohort was recruited from those attending memory clinics and approximates to the current national prevalence rates for minority ethnic participants. The limited number of minority ethnic participants precluded us from undertaking an analysis which differentiated between groups. We fully acknowledge the limitations of our approach and that differences between the groups in our samples, predominantly Indian, Pakistani, Bangladeshi, Black African and Caribbean, may be as significant as those found between these groups combined and white participants. However our exploratory study findings support the development of further studies with much larger sample sizes. This will enable us to examine variations within and between populations and combine key identity characteristics to look at, for example, how ethnicity, gender and class intersect to influence quality of life, wellbeing and life satisfaction.

The characteristics of the matched sample of people with dementia was predominantly male (80%), aged 75 years and older (69%) and diagnosed within the previous 12 months (68%). A third of carers were aged 65 years or older, 66% were female, 46% provided 10 h or more of care and 73% lived with person they cared for. Compared with the overall cohort, our matched sample of people with dementia show some nuanced differences having a higher percentage of males (58% IDEAL cohort, 80% matched groups) and the married (75% IDEAL cohort, 90% ethnic minority and 83% white matched groups) than the overall cohort. For carers, the matched samples had a higher percentage of carers under 65 years of age (66% vs. 31%) and those resident in more deprived areas (53% vs. 16%) than the overall IDEAL caregivers cohort. We fully appreciate that our matched sample sizes are small, which limits our statistical power and the inferences that can be drawn from our study. It is entirely plausible that there may be differences in other measures that our study could not detect because of limited statistical power. This limitation is not unique to our study,^20^, ^46^, ^47^ and nor is the analysis combining data from different minority ethnic groups because of limited numbers.^20^

Our analysis demonstrated that people with dementia from minority groups had lower quality of life and higher levels of loneliness than their white peers. Carers from minority ethnic groups had markedly worse scores on the specific caregiving measures (strain, relationship quality and role captivity). Data with which to compare our findings is sparse. We were unable to find previous use of the WHO‐5, life satisfaction, Lubben social isolation index, de Jong Gierveld loneliness measure or quality of life (QoL‐AD for people with dementia and WHOQoL‐BREF for carers) or carers' role indicators (stress, role captivity and relationship quality) with ethnically diverse populations of people with dementia or carers. There is some evidence supporting the disadvantaged position of minority ethnic older people and carers relative to their white peers in terms of wellbeing and quality of life.^46^, ^47^ The de Jong Gierveld loneliness measure has been used successfully with diverse groups of older people in the UK reporting higher loneliness among minority ethnic participants compared with the general population. Notably mean scores for both groups of carers exceeded the loneliness threshold score of 2, highlighting their vulnerability to loneliness.^48^ This lack of comparative data highlights the need to undertake primary research with these groups to establish ‘population norms’ to contextualise our findings for people with dementia and their carers.

## 5 CONCLUSION

Our findings suggest that, in comparison with their white peers, people with dementia from minority groups in the UK experience lower quality of life and higher loneliness and carers higher carer stress, lower relationship quality and higher role captivity. The lack of robust comparable data for this population, with or without dementia, suggests that there is a clear need to establish baseline levels of wellbeing for older people from ethnic minority groups with (and without) dementia and for carers. Our exploratory study provides a starting point for further research with larger samples to build upon our findings, facilitate analysis of the experiences of specific minority ethnic groups, consider variations withing groups and examine the factors contributing to these inequities.

## AUTHOR CONTRIBUTIONS

IDEAL investigators Christina R. Victor, Fiona E. Matthews, and Linda Clare contributed to all aspects of the IDEAL cohort including design, supporting the conduct of field work, and data acquisition, and developed the original idea for this study. Laura D. Gamble and Fiona E. Matthews conducted the study analyses and provided comments on the draft of the manuscript. Christina R. Victor draughted the manuscript. All authors provided comments on the draft of the manuscript and approved the version to be published.

## CONFLICT OF INTEREST STATEMENT

The authors report no conflicts with any product mentioned or concept discussed in this article.

## STUDY REGISTRATION

Improving the experience of Dementia and Enhancing Active Life: living well with dementia. The IDEAL study’ was registered with UKCRN, registration number 16593. Improving the experience of Dementia and Enhancing Active Life: a longitudinal perspective on living well with dementia. The IDEAL‐2 study was registered with UKCRN, registration number 37955.

## Supporting information

Supporting Information S1

Supporting Information S2

## Data Availability

IDEAL data were deposited with the UK data archive in April 2020. Details of how to access the data can be found here: https://reshare.ukdataservice.ac.uk/854317/.
