# Supplementary material for: Living well with dementia: An exploratory matched analysis of minority ethnic and white people with dementia and carers participating in the IDEAL programme
Source: Int J Geriatr Psychiatry. 2024 Jan 5;39(1):e6048. doi: 10.1002/gps.6048 (PMC10952883; doi:10.1002/gps.6048)

Supplementary Figure 1. Each round of matching for the 3 controls (white) to each case (ethnic minority) for A) people with dementia and B) caregivers

People with dementia


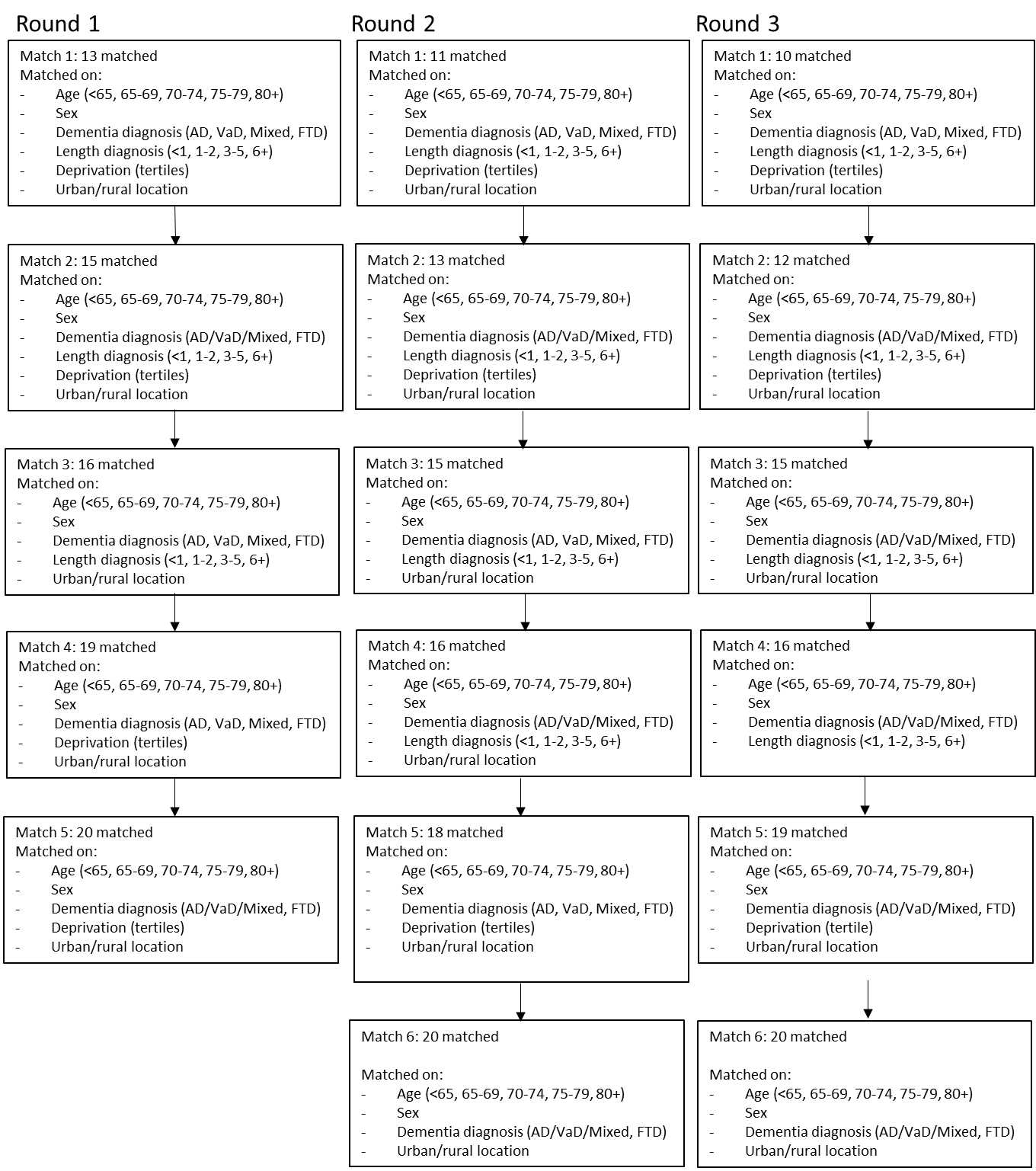


Carers


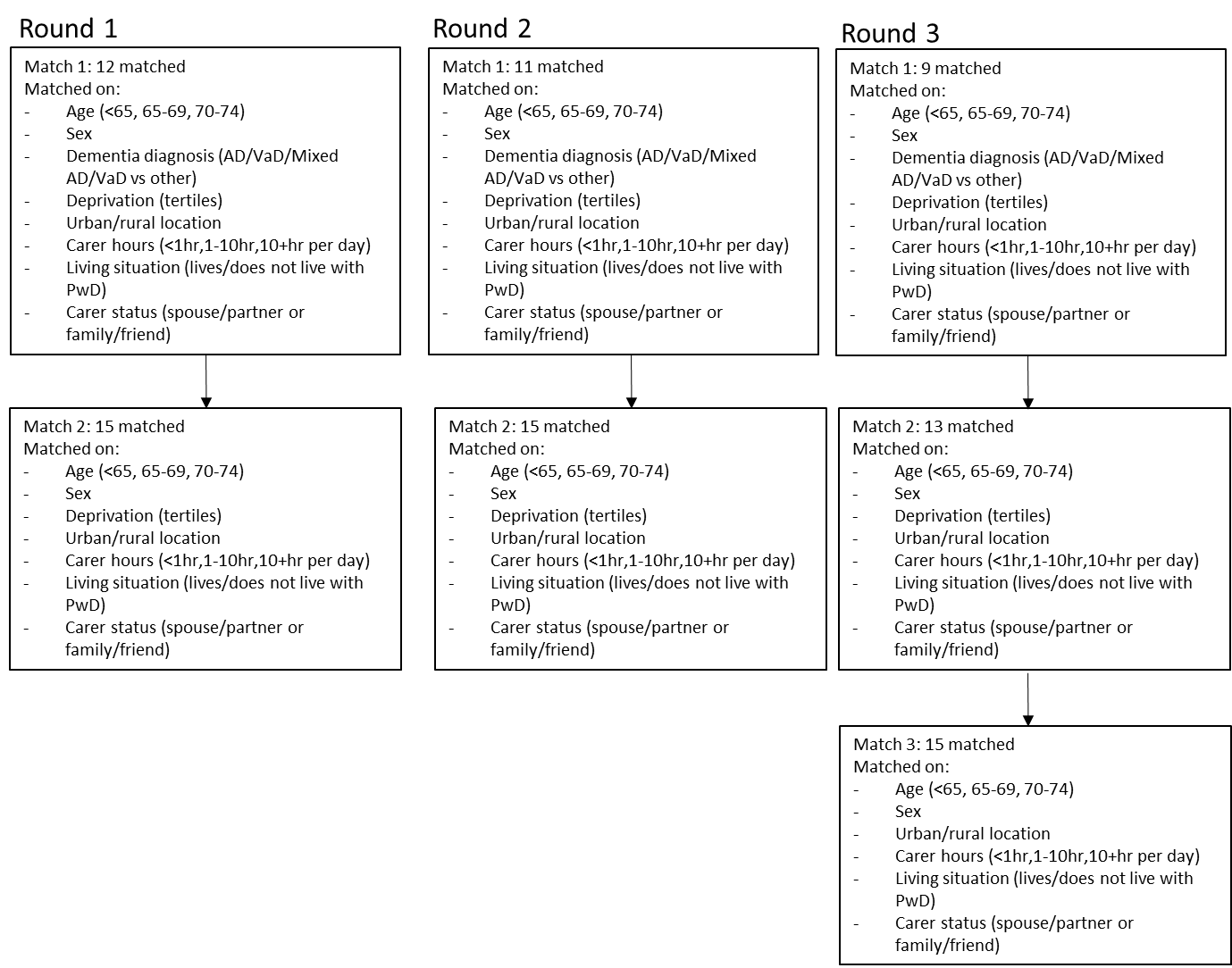

Supplement: Supplementary file 1 — Supporting Information S1 [file GPS-39-0-s001.docx]
