# Supplementary material for: Living well with dementia: An exploratory matched analysis of minority ethnic and white people with dementia and carers participating in the IDEAL programme
Source: Int J Geriatr Psychiatry. 2024 Jan 5;39(1):e6048. doi: 10.1002/gps.6048 (PMC10952883; doi:10.1002/gps.6048)
Supplement: Supplementary file 2 — Supporting Information S2 [file GPS-39-0-s002.docx]

Supplementary table 1

Detailed profile of minority ethnic participants

*Participants-people with dementia*

There are 20 people of ethnic minority in IDEAL ( 17 from T1, and 3 from enrichment cohort):

1 Mixed White and Asian

1 Other Mixed/multiple ethnic background (comment: Philippine/Spanish)

4 Asian/ Asian British: Indian

2 Asian/Asian British: Pakistani

1 Asian/Asian British: Bangladeshi

2 Any other Asian background (comment: 1 Asian Brazilian, 1 Lebanese)

3 Black/Black British: African

4 Black/Black British: Caribbean

1 Any other Black/African/Caribbean background (comment: Afro Caribbean)

1 Any other (comment: Indian/European)

*Participants-carers*

There are 15 people of ethnic minority in IDEAL (11 from T1, and 4 from enrichment cohort):

2 Mixed white & black: Caribbean

1 Mixed white and black: African

1 Other Mixed/multiple ethnic background (comment: New Zealand Māori)

2 Asian/ Asian British: Indian

2 Asian/Asian British: Pakistani

5 Any other Asian background (comment: 1 Thai, 2 Malaysian, 1 Japanese, 1 Filipino)

1 Black/Black British: African

1 Black/Black British: Caribbean
